# Stimulating Clinical Neuroscience Education: A Pilot Study of an Immersive Simulation Curriculum for Psychiatry Residents

**DOI:** 10.7759/cureus.99952

**Published:** 2025-12-23

**Authors:** Yelu Zhang, Daphne Ying, Shyam Akula, Yevgen Chornenkyy, Maria Alino-Dies, Matcheri Keshavan, Paulo Lizano

**Affiliations:** 1 Psychiatry, Beth Israel Deaconess Medical Center, Harvard Medical School, Boston, USA; 2 Neurology, University of California San Francisco, San Francisco, USA; 3 Pathology, Beth Israel Deaconess Medical Center, Harvard Medical School, Boston, USA

**Keywords:** clinical neuroscience, medical education, neuroscience education, psychiatry residency training, simulation

## Abstract

Objective

The authors evaluate the feasibility and short-term learner perceptions of a simulation-based curriculum for teaching clinical neuroscience topics to psychiatry residents, as part of a pilot educational study conducted during the 2023-2024 academic year.

Methods

A repertoire of simulation-based case scenarios was developed and implemented in this pilot study for 40 adult psychiatry residents between postgraduate years 1 and 4 at a single psychiatric residency program. All available residents were included to support implementation testing rather than statistical power. Simulation-based sessions were explored alongside a self-directed learning format covering the same content. Feasibility and short-term learner perceptions were assessed using post-session survey instruments, including quantitative Likert-scale items and open-ended qualitative feedback.

Results

Both instructional formats were feasible to implement within the residency curriculum and were associated with positive short-term learner perceptions, including increased self-reported interest and confidence following participation. Residents who participated in simulation-based sessions reported higher perceived clinical applicability of the material. Qualitative feedback suggested that simulations were engaging and supported the application of concepts to clinical scenarios.

Conclusion

This pilot study demonstrates the preliminary trends of implementing simulation-based clinical neuroscience teaching within psychiatry residency training. The primary contribution of this work is to inform curriculum design, evaluation strategies, and implementation logistics for future larger-scale, longitudinal studies.

## Introduction

Advances in the understanding of brain mechanisms behind psychiatric disorders have led to the formation of “clinical neuroscience” as a discipline within psychiatry [1,2]. Research and technological advancements have led to a better understanding of the underlying pathophysiology of psychiatric symptoms, new approaches that challenge conventional diagnostic and prognostic frameworks, and the development of novel treatment strategies [3-5]. Today’s practicing psychiatrists must have a command of this growing knowledge base and be able to apply this information in their clinical practice, with the goal of providing the best patient care and treatment [4-7]. Thus, it is important for psychiatry trainees to receive adequate clinical neuroscience teaching during their training [8].

In the context of psychiatry residency training, clinical neuroscience refers to an educational framework that integrates knowledge of neurology, neuropsychiatry, neurodiagnostic testing, and relevant neuroscience with clinical reasoning, diagnostic formulation, and treatment planning across psychiatric disorders, with an emphasis on translating this knowledge into patient-centered care [9,10]. Numerous efforts have been made to improve clinical neuroscience education in psychiatry [9,10]. This includes updated resident training guidelines by the Accreditation Council for Graduate Medical Education (ACGME) [9,10], the creation of research opportunities to support trainees interested in clinical neuroscience research [4], the implementation of clinical neuroscience-focused teaching curricula [11], and the development of online educational platforms [12,13]. Despite such efforts, many issues remain in clinical neuroscience education in psychiatry, such as a lack of teaching strategies that effectively help trainees retain neuroscience knowledge and apply it to clinical practice [5,7]. Additionally, there is less interest in learning about clinical neuroscience due to its perceived complexity, and not enough teachers with the time and/or expertise to teach such topics [5]. 

Improvements are needed so that clinical neuroscience is taught in an interesting, engaging, and clinically relevant manner. A potential solution involves the use of active learning approaches, which focus on engaging learners in a student-centered learning process and fostering learning through processes of engagement, reflection, dialogue, and collaboration [14,15]. 

Within the realm of active learning, the use of simulations is a strategy that can provide excellent experiential and patient-centered learning experiences. First used in fields such as military and aviation, simulation is defined as “a situation in which a particular set of conditions is created artificially in order to study or experience something that is possible in real life” [16,17]. Types of simulation can include informal role-playing, use of trained simulated patients (SPs), full-body simulators, mannequins, or virtual technology (such as virtual reality, augmented reality, and mixed reality platforms) [16,18]. The use of simulation methods in education is rooted in adult learning theory, allowing learners to apply prior knowledge in new scenarios, as well as obtain new knowledge through observation, perception, and modeling of behavior [19]. 

Currently, there are limited reports related to the use of simulations for clinical neuroscience education in psychiatric residency training, aside from the use of role-playing. Additionally, there is an absence of investigations evaluating the effectiveness of such methods, despite their potential to improve learners' ability to apply neuroscience principles in clinical practice. To begin addressing this gap, we conducted a pilot study to examine the impact of a simulation-based teaching approach. In this article, we describe (1) the implementation of a case-based simulation teaching curriculum to teach clinical neuroscience across all four years of psychiatry residency training at a single residency program, and (2) the evaluation of the aforementioned curriculum on learner reaction and attitudes by comparing the results of learners who participated in simulations to those who participated in a self-directed learning method. We hypothesize that residents who participate in simulations (“Sim” group) will have higher interest in clinical neuroscience topics and higher confidence to manage clinical neuroscience scenarios, compared to those who participate in an alternative learning method (“No-Sim” group) by completing self-directed learning materials. Given the pilot nature of this study, our primary aim is to examine short-term attitudinal outcomes rather than to establish definitive educational effects.

Preliminary data from this study were presented in poster format at the Carl J. Shapiro Center Medical Education Day in Boston, USA, in June 2024. Preliminary data were presented in presentation format at the Association of Academic Psychiatry Annual Meeting in Washington, D.C., USA, in September 2024.

## Materials and methods

Ten clinical scenarios were developed by the study authors, Y.Z. and M.A.D. Each scenario focused on a clinical patient presentation that contained salient neuroscience themes. Scenario topics and content were selected, developed, and reviewed in consultation with senior psychiatry faculty members who have expertise in clinical neuroscience (P.L. and M.K.). Scenarios occurred in different clinical settings (i.e., inpatient, emergency room, or outpatient), with five scenarios tailored toward junior residents (postgraduate year (PGY) 1 and 2) and five scenarios tailored toward senior residents (PGY 3 and 4). Topics were chosen to represent high-yield conditions commonly encountered in clinical psychiatry and requiring integration of neuroscience knowledge for accurate diagnosis and management. Scenarios for junior residents included (1) cognitive impairment, (2) Wernicke-Korsakoff syndrome, (3) catatonia, (4) language disorders, and (5) autoimmune encephalitis. Scenarios for senior residents included (1) brain injury sequelae, (2) normal pressure hydrocephalus, (3) Parkinson’s and related diseases, (4) Huntington’s disease, and (5) functional neurological disorder. This selection reflects a developmental progression - from foundational understanding of brain-behavior relationships and medical causes of psychiatric symptoms, often emphasized in acute and inpatient settings during the junior years of residency training, to advanced application of clinical neuroscience in the longitudinal management of complex and chronic conditions requiring nuanced clinical reasoning, which is more characteristic of senior years of training.

Each scenario was simulated using a trained SP, and, when necessary, a confederate - a trained individual acting as a family member or collateral contact. Simulations were conducted at the hospital’s dedicated simulation center, equipped with resources including vital sign machines, hospital beds, and gurneys to simulate realistic clinical environments. Each scenario was adapted into a facilitator guide with detailed instructions for easy adaptation by facilitators and SPs for use in various educational settings, regardless of access to a simulation center. Additionally, a self-directed learning “slide deck” was created using Microsoft PowerPoint (Microsoft® Corp., Redmond, WA, USA) for each scenario, which was used to relay relevant learning points.

A mixed-methods pilot study was conducted at Beth Israel Deaconess Medical Center and included all available psychiatry residents enrolled during the study period (N = 40; 10 residents per PGY 1-4). All residents were eligible to participate to maximize feasibility testing and ecological validity. No formal exclusion criteria were applied. The study received Institutional Review Board exemption as educational research and is intended as a preliminary study of the feasibility and short-term outcomes of the curriculum, rather than to establish definitive efficacy.

**Figure 1:**
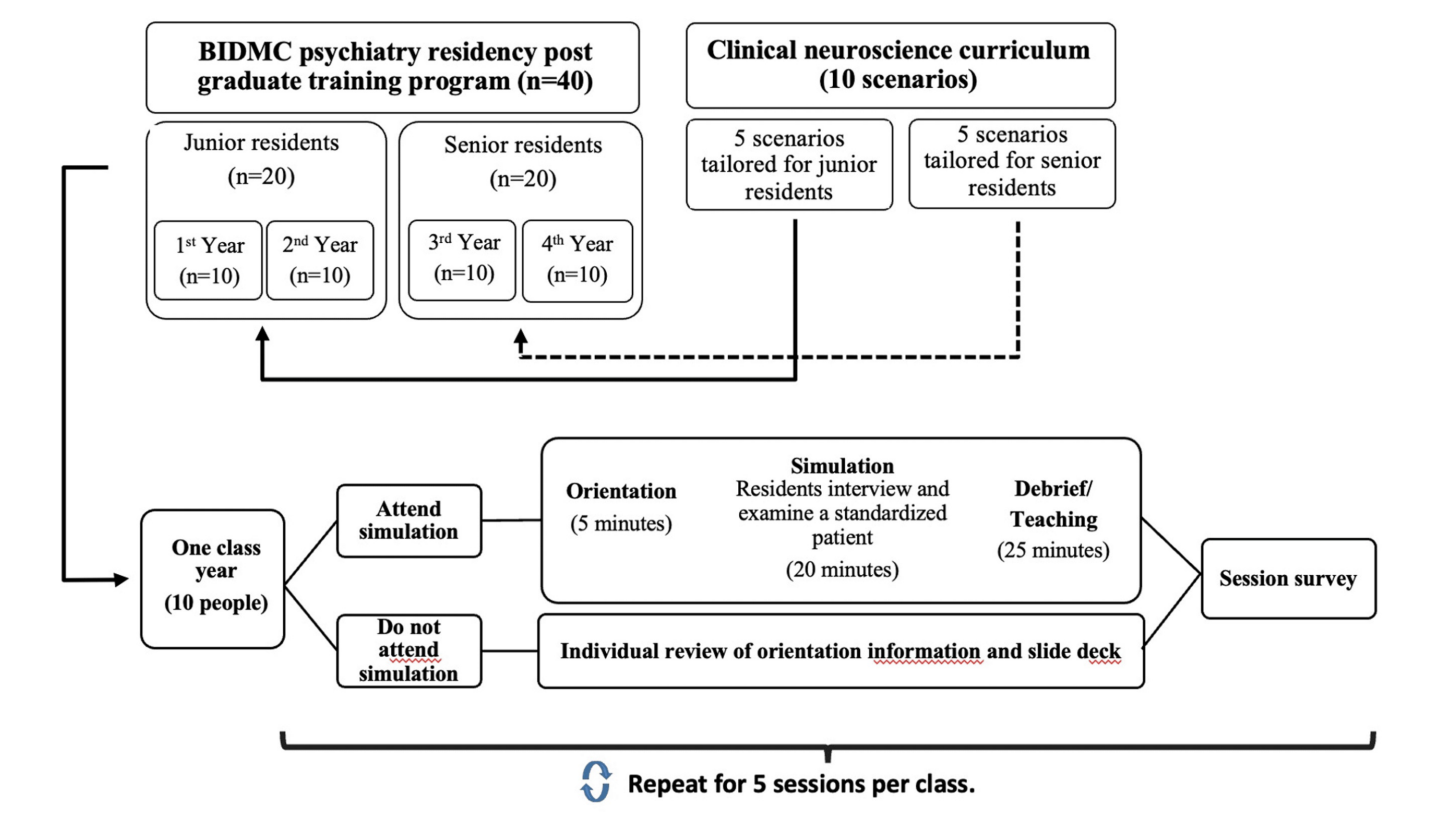
Summary of study design Figure created by Y.Z. with Microsoft PowerPoint.

Between December 2023 and June 2024, a five-session-long teaching curriculum was implemented for each residency class year in coordination with the residency program directors. One case scenario was presented at each session, with junior residents completing five scenarios and senior residents completing a different set of five scenarios. At each session, approximately half of the residents in the class (about five learners) were randomized using an online list generator (random.org) to participate in simulations (“Sim” group). In these sessions, residents interviewed and evaluated an SP, followed by a facilitated group discussion led by one or two facilitators who were senior residents or faculty members (P.L., M.K., Y.Z., and M.A.D.). The remaining residents were randomized to the “No-Sim” group and completed the self-directed learning materials covering the same content. Learners who were not able to attend a simulated session for any reason (i.e., sickness or vacation) also received the self-directed learning materials. Please see Figure 1 for a summary of the study design.

After each session, participants were invited to complete a voluntary survey distributed via email and accessible through a QR code, assessing the preliminary perceptions of the teaching session using both quantitative and qualitative modality-based questions. Participants first indicated whether they had completed the simulation or the self-directed learning activity. To ensure survey completeness and design integrity, a third option, “I did not participate in either learning activity,” was included as a safeguard to capture any respondents who might have accessed the survey inadvertently or outside of their assigned group. This option was included to ensure response validity and was not intended as an analytic group. Quantitative survey items assessed short-term learner perceptions using five-point Likert scales, including (1) interest in the topic before and after the session, (2) confidence in managing the clinical condition before and after the session, and (3) perceived clinical applicability of the material. A retrospective pre-post design was used for interest and confidence measures to reduce response-shift bias. Survey content validity was established through expert review (Y.Z., D.Y., P.L., and S.A.). For each session, pre- and post-session interest and confidence levels were averaged separately within the “Sim” and “No-Sim” groups. Changes in pre- to post-session ratings were analyzed using the Wilcoxon paired signed-rank test. For the perceived clinical applicability item, responses were compared between the “Sim” and “No-Sim” groups using the Mann-Whitney U test. The statistical analyses were performed for exploratory purposes to identify preliminary trends. GraphPad Prism (GraphPad Software, San Diego, CA, USA) was used for statistical analysis. 

At the end of every survey, participants were invited to provide qualitative feedback through free-text responses. These comments were descriptively analyzed to identify common themes and patterns by Y.Z. and D.Y. This qualitative component was considered a key element of the pilot study, providing complementary evidence of learner perceptions and informing future curriculum refinement. Please refer to Appendix A for a sample survey.

## Results

A total of 79 responses were collected from participants who attended simulations, and 38 responses were collected from those who completed self-directed learning sessions. No respondents selected the “did not participate” option, confirming that all survey respondents engaged in one of the two learning modalities. The anticipated response rate was approximately 100 per group, corresponding to a 79% and 38% response rate for the “Sim” and “No-Sim” groups, respectively. This estimate was derived from the total number of survey opportunities across the curriculum (10 residents per class × 5 sessions × 4 class years = 200 responses, with approximately 100 randomized to each group). Please see Table 1 for a detailed breakdown of response rates among individual sessions.

**Table 1:** Breakdown of number of responses by scenario

Training level	Scenario topic	Number of responses (_/10) (Sim)	Number of responses (_/10) (No Sim)
Junior residents (PGY1 and PGY2)	Cognitive impairment	9	5
	Wernicke-Korsakoff	9	1
	Catatonia	8	4
	Language disorders	7	0
	Autoimmune encephalitis	6	1
Senior residents (PGY3 and PGY4)	Brain injury and sequelae	7	8
	Normal pressure hydrocephalus	9	6
	Parkinson’s and related diseases	9	3
	Huntington’s disease	8	7
	Functional neurological disorder	7	3
Total number of responses (%)		79/100 (79%)	38/100 (38%)

**Figure 2:**
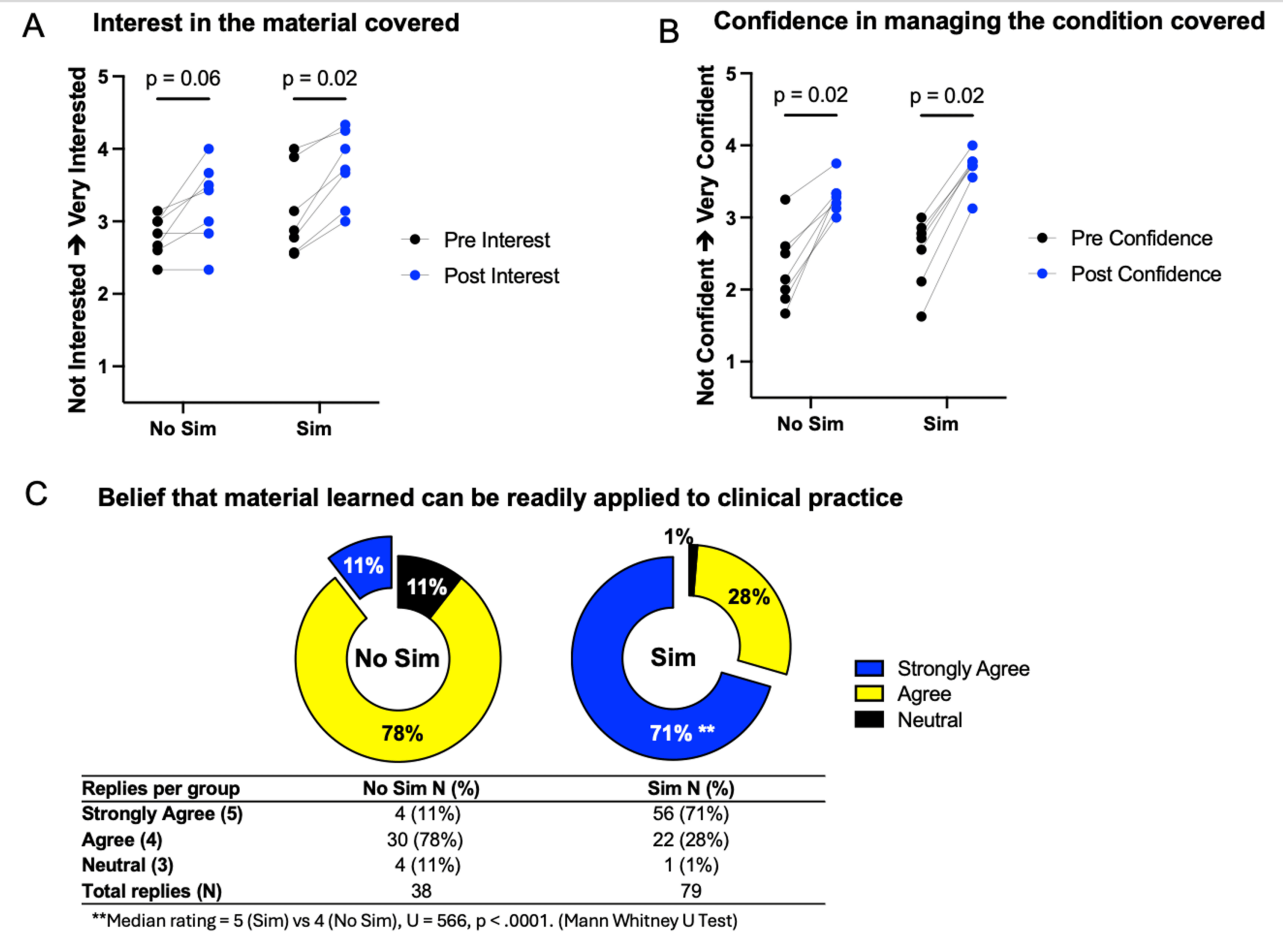
Summary of quantitative data results (A): Comparison of pre- and post-interest levels in Sim and No-Sim groups. Y-axis: Each data point represents a curriculum topic and reflects the mean response of all participants within the respective group. Y-axis: 1 = not interested at all, 2 = slightly interested, 3 = somewhat interested, 4 = very interested, 5 = extremely interested. (B): Comparison of pre- and post-confidence levels in Sim and No-Sim groups. Y-axis: Each data point represents a curriculum topic and reflects the mean response of all participants within the respective group. Y-axis: 1 = not confident at all, 2 = slightly confident, 3 = somewhat confident, 4 = very confident, 5 = extremely confident. (C): Participants’ beliefs regarding the clinical applicability of their learning session. Responses were measured using a five-point Likert scale (1 = strongly disagree, 2 = disagree, 3 = neutral, 4 = agree, 5 = strongly agree).

Of the 10 topics included in the curriculum, seven were included in the exploratory analyses of interest and confidence, while three were excluded due to insufficient numbers of responses. The average response of all participants within the respective group was calculated per session. Based on these descriptive analyses, both the "Sim" and "No-Sim" groups demonstrated overall increases in interest and confidence following the educational sessions. Interest in learning the topic was measured using a five-point Likert scale (1 = not interested at all, 2 = slightly interested, 3 = somewhat interested, 4 = very interested, and 5 = extremely interested), and confidence in managing the clinical condition covered in each teaching session was assessed using a five-point Likert scale (1 = not confident at all, 2 = slightly confident, 3 = somewhat confident, 4 = very confident, and 5 = extremely confident). As shown in Figure 2A, within-group changes in interest levels were explored using non-parametric tests and were more pronounced in the "Sim" group (Wilcoxon signed-rank test, W = 28, p < 0.05) compared with the "No-Sim" group (W = 25, p = 0.06). Similarly, changes in confidence levels were observed in both groups (Wilcoxon signed-rank test, p < 0.05; Figure 2B). Given that the datasets were unpaired and response rates differed between groups, between-group comparisons of the magnitude of pre- to post-session change in interest or confidence were not performed. Perceived clinical applicability of the educational content was assessed using a five-point Likert scale (1 = strongly disagree, 2 = disagree, 3 = neutral, 4 = agree, and 5 = strongly agree), with participants in the "Sim" group reporting higher ratings of clinical applicability compared with the "No-Sim" group (Mann-Whitney U test, U = 566, p < 0.0001; Figure 2C).

**Figure 3:**
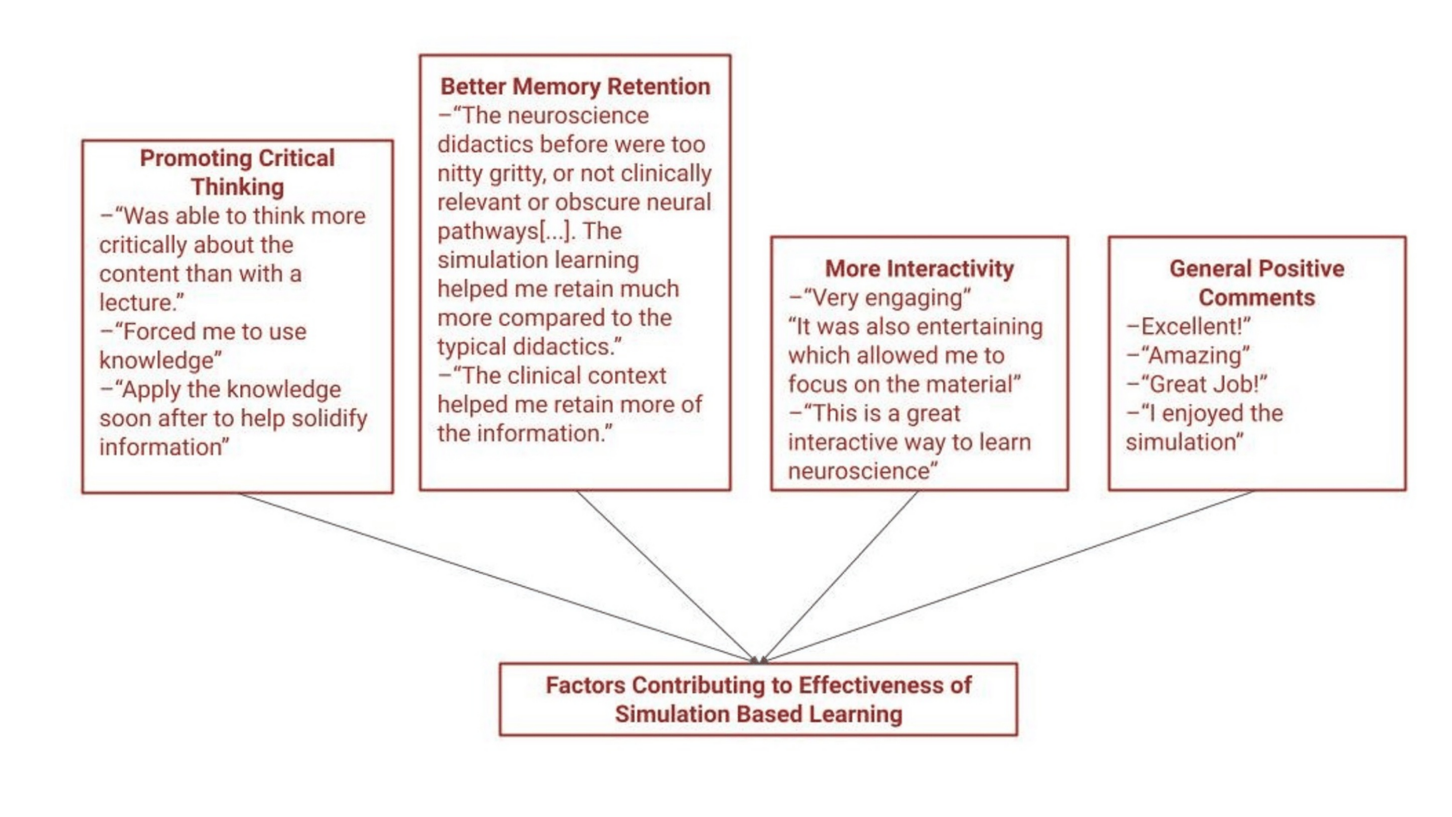
Post-survey qualitative comments

Twelve free-text comments were collected from surveys conducted across all teaching sessions. Given the limited number of responses, descriptive rather than thematic analyses were performed. Nearly all comments pertained to the simulation component of the curriculum, with only one providing constructive feedback on the self-directed learning materials. Overall, learners expressed positive impressions of the simulation sessions, noting that they promoted critical thinking (three comments), enhanced memory retention (two comments), were interactive (two comments), and were a fun and engaging learning experience (four comments) (Figure 3).

## Discussion

Simulations have been utilized in psychiatry education in areas including, but not limited to, suicide risk assessment, therapy techniques, substance use disorders, anxiety and depression management, and empathy-building [17]. Prior meta-analyses have demonstrated that simulations in psychiatry training significantly enhance immediate post-test scores for knowledge, attitudes, skills, and behaviors compared with the control groups [17]. Additionally, significant differences have also been reported for patient outcomes and skills- and behavior-related outcomes at three-month follow-up [17]. In this study, a novel simulation-based clinical neuroscience curriculum utilizing SPs was implemented across all four years of psychiatry residency training at a single residency program. To our knowledge, this is the first study to investigate the use of a full simulation-based curriculum specifically for clinical neuroscience education in psychiatry. 

Overall, this pilot study yielded predominantly positive short-term learner perceptions, with increases in post-session interest and confidence observed across both teaching modalities. These findings suggest that participation in a structured clinical neuroscience curriculum - regardless of format - was perceived as beneficial over time. Within-group exploratory analyses indicated a greater increase in interest among learners who participated in simulations, while confidence levels increased in both learners who participated in simulations and those who did not. Given the pilot nature of the study and the absence of formal between-group comparisons, these observations should be interpreted as preliminary trends. In addition, simulations were associated with higher perceived clinical applicability of the material, with a greater proportion of learners in the "Sim" group strongly endorsing clinical relevance. Although the qualitative feedback collected was limited, it provided useful insights into learner experience - highlighting themes that simulations fostered critical thinking, memory retention, interactivity, and enjoyment in the learning process.

Taken together, our findings offer preliminary evidence supporting the feasibility and acceptability of the use of simulations in clinical neuroscience education, as well as reinforcing the broader value of simulation as an effective educational tool [17]. One of the contributions of this pilot study is in testing curriculum logistics and preliminary learner perceptions, thereby informing the design of future larger-scale and longitudinal studies. In particular, simulations may be a promising method to address some of the well-known challenges of clinical neuroscience education in psychiatry training, such as perceived lack of clinical relevance and limited learner engagement [5]. This merits further investigation in adequately powered studies. 

While the creation of novel neuroscience curricula has been previously reported in psychiatry [11-13], simulations may serve as an additional method to diversify teaching approaches. Grounded in experiential learning principles, they can help learners apply knowledge to real-life scenarios [17]. This approach may complement or improve upon traditional methods, like case conferences or journal clubs, which, while valuable, are often focused on passive learning processes and are subsequently less engaging for learners [20]. 

Although the use of simulations has many benefits, there are also some nuances to be considered. These include significant required learner engagement (which may cause emotional pressure or discomfort), high resource and time demands for facilitators, and variability of simulations or SPs in authentically depicting the complex set of cognitive, emotional, and psychological features associated with psychiatric conditions [18]. Thus, the use of simulations in clinical neuroscience and/or other aspects of education in psychiatry requires further investigation. In our study, we focused exclusively on the use of SPs, given their ability to better depict complex cognitive and affective features compared with other simulation types, such as mannequins or high-fidelity simulators. However, further studies exploring and comparing the use of different simulation methods may be beneficial.

This study has several limitations consistent with its pilot design. Implementation occurred at a single urban, academic residency program. This introduces potential bias toward the results collected, which may differ compared with residents training at other institutions. Expanding the curriculum to other training programs will improve generalizability and assess the impact of institutional differences. Another limitation of our study is the significant data attrition in survey responses, particularly for the “No-Sim” group. This highlights challenges in engaging participation in optional survey evaluations. Furthermore, we cannot rule out a placebo-like effect that may have contributed to learners' perceptions of benefit from teaching [21]. Finally, the survey instruments were designed to capture short-term perceptions and did not assess longer-term outcomes, such as knowledge retention or changes in clinical practice. These limitations underscore the role of this study as a foundational step toward a more comprehensive evaluation in the future.

## Conclusions

This pilot study suggests that simulation-based approaches may be a feasible and beneficial method for clinical neuroscience education in psychiatry residency training. The observed findings are preliminary, indicating that simulation-based teaching may be associated with increased learner interest, confidence, and perceived clinical applicability of clinical neuroscience concepts. These results should be interpreted in the context of the pilot nature of the study and the focus on short-term attitudinal outcomes rather than definitive measures of educational effectiveness. This work primarily informs the design of future larger-scale and longitudinal studies needed to more rigorously evaluate educational impact, knowledge retention, and effects on clinical practice. Future efforts may also focus on continued curriculum refinement, expansion to additional learner groups or training programs, and the collection of longitudinal outcomes to better assess impacts over time.

## Appendices

Appendix A - Simulations post-survey

(1) I attended the simulation session on ___ (wording adjusted based on specific topic covered in simulation)

○ Yes ○ No, but I reviewed the slide deck instead ○ No, I did not participate in any learning activity

Simulation

(2) What role did you take in the simulation?

○ Interviewing or examining the patient ○ Observing, taking notes, or other roles

(3) In the following situations, please rate how confident you feel in your ability to provide the necessarily management for a patient presenting with ___ (wording adjusted based on specific topic covered in simulation)

**Table 2:** Please rate how confident you feel in your ability to provide the necessarily management for a patient

	Extremely not confident	Slightly confident	Some what confident	Very confident	Extremely confident
BEFORE attending the simulation					
AFTER attending the simulation					

(4) In the following situations, please rate your level of interest in learning about ___ (wording adjusted based on specific topic covered in simulation)

**Table 3:** Please rate your level of interest in learning

	Not interested at all	Slightly interested	Moderately interested	Very interested	Extremely interested
BEFORE attending the simulation					
AFTER attending the simulation					

(5) Use the following scale to rate your impression of how effective the simulation facilitator was in facilitating the following aspects of the simulation

**Table 4:** Please rate your impression of how effective the simulation facilitator was in facilitating the following aspects of the simulation

	Very ineffective	Somewhat ineffective	Neither effective nor ineffective	Somewhat effective	Very effective
The instructor maintained an engaging learning experience					
The instructor structured the debriefing in anorganized fashion					
The instructor provided indepth discussion that led me to reflect on my performance					

(6) Please select how much you agree with the following statements

**Table 5:** Please select how much you agree with the following statements

	Strongly disagree	Disagree	Neutral	Agree	Strongly agree
After completing this session, I learned material that can be readily applied to my clinical training/practice					
This learning session was interesting					

(7) Please provide any comments about how you felt about your simulation experience, including any positive or negative experiences, suggestions for future, and anything you have learned from the experience: _________________________

Self-Directed Learning

(8) In the following situations, please rate how confident you feel in your ability to provide the necessarily management for a patient presenting with ___ (wording adjusted based on specific topic covered)

**Table 6:** Please rate how confident you feel in your ability to provide the necessarily management for a patient

	Extremely not confident	Slightly confident	Some what confident	Very confident	Extremely confident
BEFORE attending the simulation					
AFTER attending the simulation					

(9) In the following situations, please rate your level of interest in learning about ___ (wording adjusted based on specific topic covered)

**Table 7:** Please rate your level of interest in learning

	Not interested at all	Slightly interested	Moderately interested	Very interested	Extremely interested
BEFORE attending the simulation					
AFTER attending the simulation					

(10) Please select how much you agree with the following statements

**Table 8:** Please select how much you agree with the following statements

	Strongly disagree	Disagree	Neutral	Agree	Strongly agree
After completing this session, I learned material that can be readily applied to my clinical training/practice					
This learning session was interesting					

(11) Please provide any comments about how you felt about your learning experience, including any positive or negative experiences, suggestions for future, and anything you have learned from the experience ____________________

Did Not Participate in Any Learning Session

(12) How confident you feel in your ability to provide the necessarily management for a patient presenting with ___ (wording adjusted based on specific topic covered)

○ Extremely not confident ○ Slightly confident ○ Somewhat confident ○ Very confident ○ Extremely confident

(13) Please rate your level of interest in learning about ___ (wording adjusted based on specific topic covered)

○ Not interested at all ○ Slightly interested ○ Moderately interested ○ Very interested ○ Extremely interested

(14) Please provide any additional comments you would like to share __________
